# Supplementary material for: Tracking of Melanoma Cell Plasticity by Transcriptional Reporters
Source: Int J Mol Sci. 2022 Jan 21;23(3):1199. doi: 10.3390/ijms23031199 (PMC8835814; doi:10.3390/ijms23031199)
Supplement: Supplementary file 1 [file ijms-23-01199-s001.zip › supplementary ijms-1542685.pdf]

International Journal of *Molecular Sciences*

MDPI

Supplementary file

Tracking of melanoma cell plasticity by transcriptional reporters

Anna Vidal[1] and Torben Redmer[2,*]

Supplementary figures

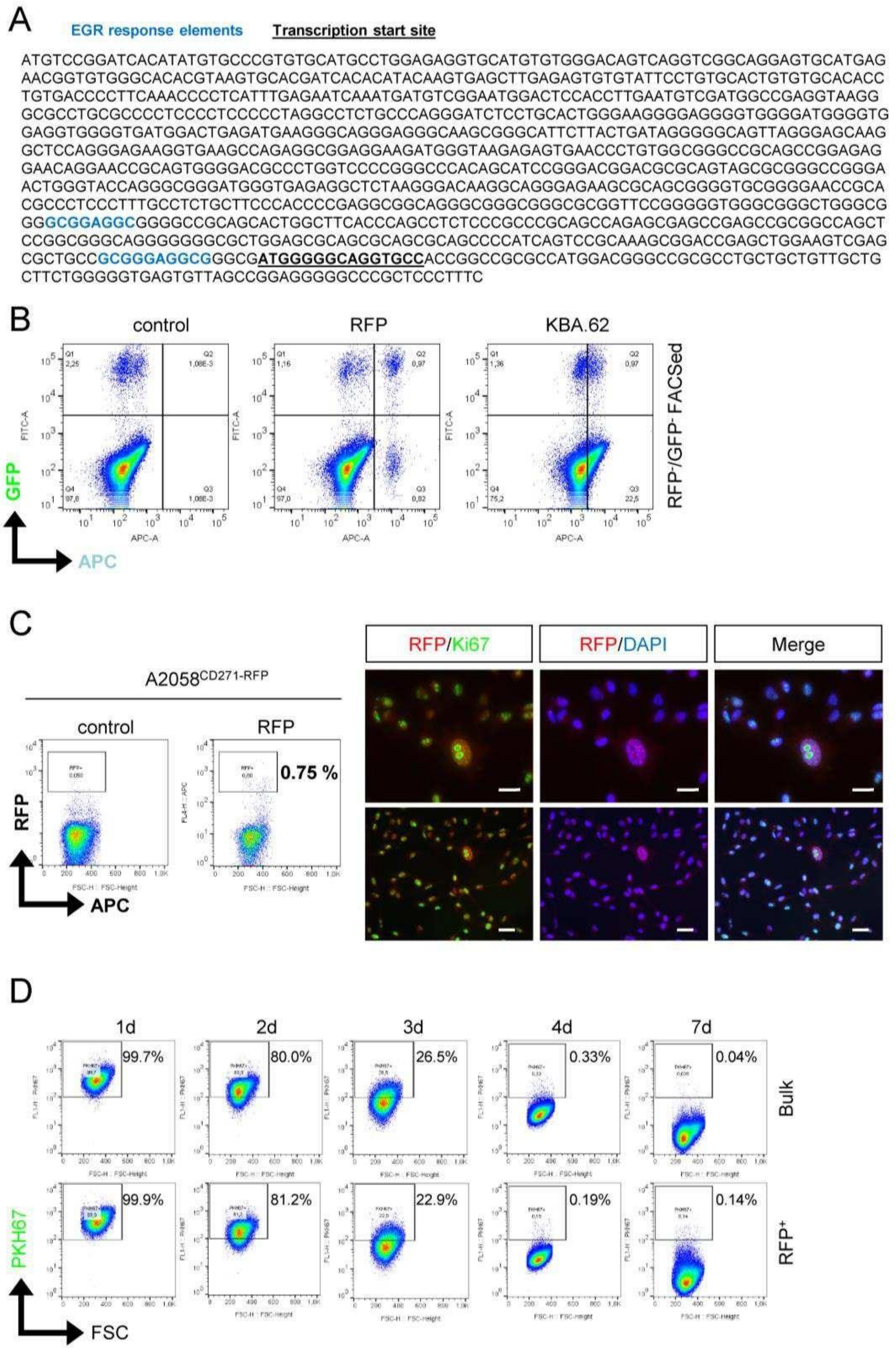

Supplementary figure 1: Assessment of the label-retaining capacity of reporter cells. A.) Sequence of a 1 kb fragment of the NGFR promoter that has been cloned into a customized RFP-reporter vector. Early growth response (EGR) elements are shown in blue. transcriptional start site is shown in bold and underlined. B.) Flow cytometric analysis of double-negative sorted dual-reporter A375 cells for levels of reporters (GFP. RFP) and KBA.62. 7d after FACS demonstrating cellular plasticity via emergence of NGFR+ from negative cells. An RFP-specific antibody was used for detection of RFP+ cells. C.) Flow cytometry and fluorescence microscopy-based detection of a minor RFP^{+} subset in A2058 cells. D.) Flow cytometric analysis of cellular subsets potentially retaining the lipophilic dye PKH67 in bulk and RFP+ cells after 7d. In (c); 10.000 and (b) and (d); 50.000 cells were recorded.

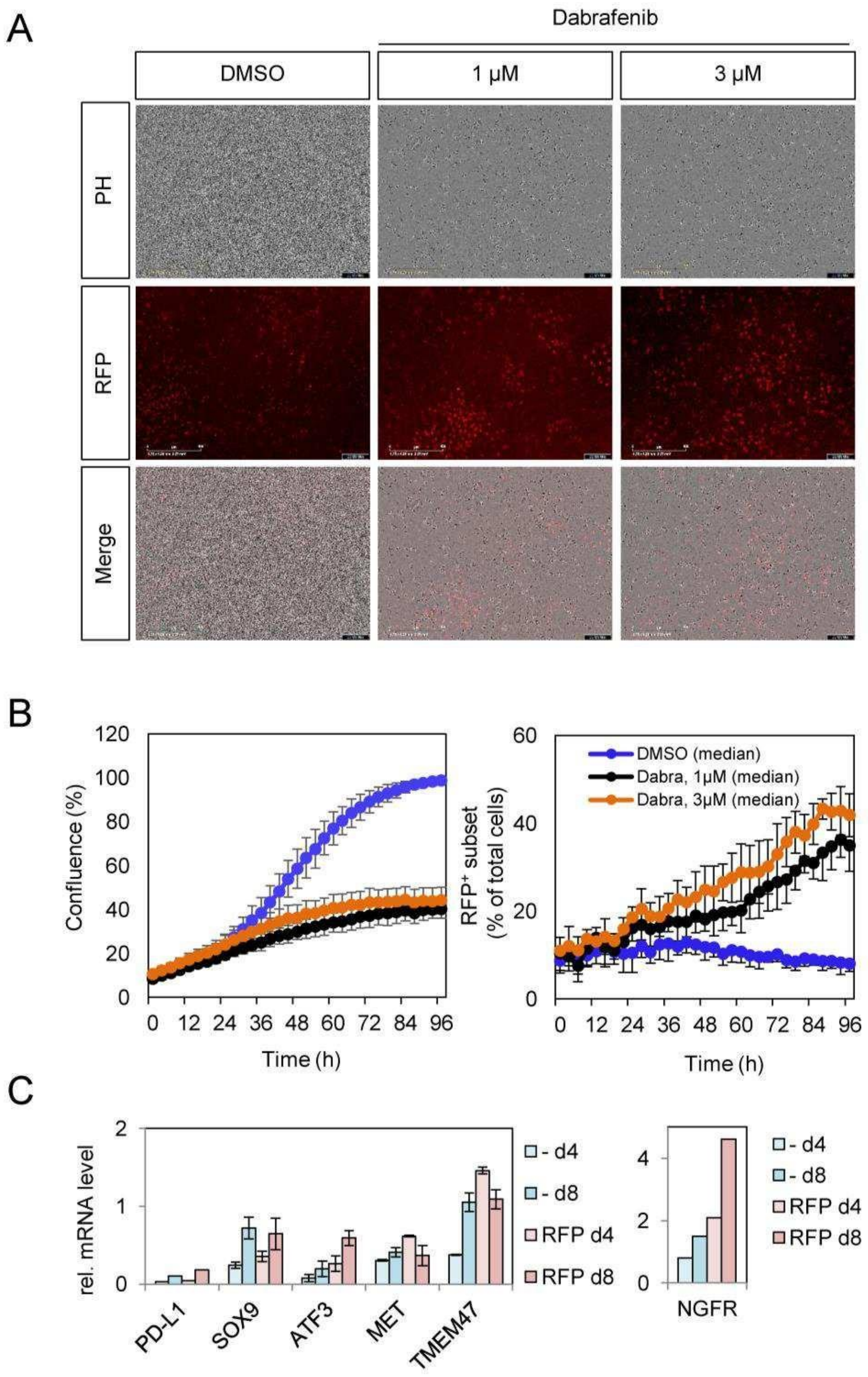

Supplementary figure 2: Phenotype switching regulates putative mediators of stemness. A.) Merged bright field and fluorescence images of A375 reporter cells indicate the increase in the number of RFP+ cells in response to dabrafenib (1μM. 3 μM. 96h) as tracked by live cell imaging. Bars indicate 400 μm. B.) Dose-response of A375 cells towards dabrafenib (1. 3 μM) as determined by changes in confluence (left panel). Live cell imaging-based quantification revealed a dose-dependent increase of RFP+ cells over time (right panel) as compared to treatment control (DMSO). (right panel). C.) qPCR of RFP+ (RFP) and RFP negative (-) sorted reporter cells. 4d and 8d after culturing for levels of PD-L1 (CD274). SOX9. ATF3. MET and TMEM47. values are depicted as mean±sdv (left panel) and levels of NGFR (right panel).

Supplementary tables

Table S1: Differentially regulated genes among RFP+ and bulk cells (A375 reporter)

ID	baseMean	log2FoldChange	pvalue	padj	Gene symbol
ENSG00000162892	1277.82	4.277	1.69E-77	2.51E-73	IL24
ENSG00000196611	1085.14	-3.830	2.15E-66	3.20E-62	MMP1
ENSG00000048052	1390.93	3.316	2.87E-55	4.27E-51	HDAC9
ENSG00000175445	528.36	4.072	7.05E-52	1.05E-47	LPL
ENSG00000154027	928.39	3.590	5.22E-51	7.77E-47	AK5
ENSG00000023839	2492.27	-2.959	2.36E-50	3.52E-46	ABCC2
ENSG00000265972	8157.13	3.110	3.10E-50	4.61E-46	TXNIP
ENSG00000113532	1497.62	-3.276	3.20E-48	4.76E-44	ST8SIA4
ENSG00000137868	1199.12	-3.132	1.93E-46	2.86E-42	STRA6
ENSG00000171051	338.83	-5.768	2.14E-45	3.18E-41	FPR1
ENSG00000156687	1701.10	-3.002	1.28E-43	1.90E-39	UNC5D
ENSG00000128340	474.44	-4.278	7.08E-43	1.05E-38	RAC2
ENSG00000149294	348.97	3.945	4.42E-41	6.57E-37	NCAM1
ENSG00000265107	278.76	-5.725	1.50E-40	2.24E-36	GJA5
ENSG00000151789	612.33	-3.053	2.43E-38	3.61E-34	ZNF385D
ENSG00000049192	3877.64	2.550	3.99E-38	5.94E-34	ADAMTS6
ENSG00000213658	238.27	-4.162	2.85E-37	4.24E-33	LAT
ENSG00000196549	3210.60	2.571	7.20E-37	1.07E-32	MME
ENSG00000183688	633.09	3.025	1.73E-36	2.57E-32	RFLNB
ENSG00000117069	831.96	2.978	6.87E-36	1.02E-31	ST6GALNAC5
ENSG00000145681	4797.28	3.010	3.40E-34	5.06E-30	HAPLN1
ENSG00000120949	336.66	-3.380	6.25E-33	9.30E-29	TNFRSF8
ENSG00000106034	990.03	3.359	7.95E-33	1.18E-28	CPED1
ENSG00000124225	1677.82	-2.464	1.41E-32	2.10E-28	PMEPA1
ENSG00000104419	6776.00	2.664	1.98E-32	2.95E-28	NDRG1
ENSG00000223638	399.47	3.216	2.14E-32	3.18E-28	RFPL4A

ENSG00000227234	11466.30	2.698	3.10E-32	4.61E-28	SPANXB1
ENSG00000147027	919.34	2.792	3.88E-32	5.76E-28	TMEM47
ENSG00000120594	1082.55	-2.458	2.72E-31	4.04E-27	PLXDC2
ENSG00000071242	361.14	3.116	6.25E-31	9.29E-27	RPS6KA2
ENSG00000148677	7803.00	2.185	8.69E-31	1.29E-26	ANKRD1
ENSG00000170989	805.34	2.629	2.88E-30	4.29E-26	S1PR1
ENSG00000184588	497.63	-2.875	9.03E-30	1.34E-25	PDE4B
ENSG00000164251	2402.96	-2.349	1.09E-29	1.63E-25	F2RL1
ENSG00000128965	1282.11	2.372	1.26E-29	1.87E-25	CHAC1
ENSG00000148513	807.25	2.433	1.20E-27	1.79E-23	ANKRD30A
ENSG00000138829	219.93	4.156	3.43E-27	5.10E-23	FBN2
ENSG00000115414	13272.86	-2.290	6.02E-27	8.95E-23	FN1
ENSG00000118971	192.38	6.589	7.28E-27	1.08E-22	CCND2
ENSG00000165507	276.72	3.052	3.85E-26	5.73E-22	DEPP1
ENSG00000100311	151.59	4.506	4.61E-26	6.86E-22	PDGFB
ENSG00000116761	816.74	2.371	5.82E-26	8.65E-22	CTH
ENSG00000204389	1583.61	-2.654	9.84E-26	1.46E-21	HSPA1A
ENSG00000134363	6678.73	2.561	1.10E-25	1.64E-21	FST
ENSG00000151376	337.39	2.896	1.22E-25	1.82E-21	ME3
ENSG00000162772	1058.53	2.238	1.23E-25	1.82E-21	ATF3
ENSG00000138759	532.66	2.764	1.63E-25	2.42E-21	FRAS1
ENSG00000164161	993.32	2.386	2.69E-25	4.00E-21	HHIP
ENSG00000184564	249.33	-3.086	7.33E-25	1.09E-20	SLITRK6
ENSG00000022556	1433.48	-2.212	1.31E-24	1.94E-20	NLRP2
ENSG00000137767	572.33	2.373	2.31E-24	3.44E-20	SQOR
ENSG00000155265	351.31	-2.688	2.89E-24	4.30E-20	GOLGA7B
ENSG00000196562	3140.58	-2.213	3.80E-24	5.66E-20	SULF2
ENSG00000229292	234.28	3.372	3.87E-24	5.75E-20	RFPL4AL1
ENSG00000122641	8933.85	1.929	4.45E-24	6.62E-20	INHBA
ENSG00000100867	192.50	-3.059	1.77E-22	2.64E-18	DHRS2
ENSG00000117318	2965.95	-2.180	2.40E-22	3.57E-18	ID3
ENSG00000162511	108.15	-5.939	2.92E-22	4.35E-18	LAPTM5
ENSG00000213949	6198.86	-2.686	3.90E-22	5.79E-18	ITGA1
ENSG00000146674	2395.98	2.019	6.01E-19	8.94E-15	IGFBP3
ENSG00000170909	258.56	2.729	5.44E-21	8.10E-17	OSCAR
ENSG00000140563	1224.32	2.018	6.22E-21	9.25E-17	MCTP2
ENSG00000204388	1451.76	-2.511	7.86E-21	1.17E-16	HSPA1B
ENSG00000109971	148411.09	-1.847	9.39E-21	1.40E-16	HSPA8
ENSG00000198573	2036.31	1.858	1.47E-20	2.19E-16	SPANXC
ENSG00000003436	2659.00	1.987	3.19E-21	4.75E-17	TFPI
ENSG00000183287	5362.01	2.108	2.48E-20	3.69E-16	CCBE1

ENSG00000125968	4248.97	-2.142	2.57E-20	3.83E-16	ID1
ENSG00000159200	3180.53	1.780	2.90E-20	4.32E-16	RCAN1
ENSG00000166033	1510.95	-2.959	3.74E-20	5.57E-16	HTRA1
ENSG00000142871	7489.34	1.801	4.63E-20	6.89E-16	CYR61
ENSG00000104327	110.31	5.402	7.35E-20	1.09E-15	CALB1
ENSG00000165118	293.02	2.482	1.18E-19	1.76E-15	C9orf64
ENSG00000198732	1830.08	1.908	1.76E-19	2.62E-15	SMOC1
ENSG00000087494	594.12	-2.098	1.87E-19	2.78E-15	PTHLH
ENSG00000151617	313.16	-2.523	2.46E-19	3.66E-15	EDNRA
ENSG00000149968	242.98	-3.462	2.62E-19	3.90E-15	MMP3
ENSG00000132274	298.21	-2.658	2.71E-19	4.03E-15	TRIM22
ENSG00000025039	787.44	1.979	2.91E-19	4.33E-15	RRAGD
ENSG00000101210	3510.02	1.909	1.84E-20	2.74E-16	EEF1A2
ENSG00000120217	1173.85	1.857	7.15E-19	1.06E-14	CD274
ENSG00000104177	1786.13	1.722	6.45E-15	9.60E-11	MYEF2
ENSG00000115902	1744.44	2.022	8.88E-19	1.32E-14	SLC1A4
ENSG00000174951	243.63	2.556	1.15E-18	1.72E-14	FUT1
ENSG00000137959	393.56	-2.563	1.67E-18	2.49E-14	IFI44L
ENSG00000074410	155.08	-4.048	2.00E-18	2.97E-14	CA12
ENSG00000243709	115.40	-4.796	2.51E-18	3.73E-14	LEFTY1
ENSG00000074416	3067.96	2.285	2.73E-18	4.06E-14	MGLL
ENSG00000107159	786.94	2.381	3.45E-18	5.14E-14	CA9
ENSG00000114315	410.84	-2.099	6.29E-18	9.36E-14	HES1
ENSG00000197928	176.73	2.867	7.41E-18	1.10E-13	ZNF677
ENSG00000163347	90.67	4.827	7.63E-18	1.14E-13	CLDN1
ENSG00000174600	87.71	-5.051	9.03E-18	1.34E-13	CMKLR1
ENSG00000170579	134.56	3.194	1.06E-17	1.57E-13	DLGAP1
ENSG00000144821	753.43	-1.914	1.13E-17	1.68E-13	MYH15
ENSG00000075340	2507.09	1.778	1.69E-17	2.51E-13	ADD2
ENSG00000148344	1037.13	-2.309	1.91E-17	2.84E-13	PTGES
ENSG00000116183	298.83	2.306	2.02E-17	3.01E-13	PAPPA2
ENSG00000130766	1869.56	1.777	2.64E-17	3.93E-13	SESN2
ENSG00000166342	1225.85	1.804	2.80E-17	4.17E-13	NETO1
ENSG00000106123	91.64	-3.944	3.48E-17	5.17E-13	EPHB6
ENSG00000214944	3333.85	1.347	1.30E-11	1.93E-07	ARHGEF28
ENSG00000077942	111.16	3.583	4.45E-17	6.61E-13	FBLN1
ENSG00000143195	352.99	-2.160	6.04E-17	8.99E-13	ILDR2
ENSG00000104368	7752.59	1.777	6.18E-17	9.19E-13	PLAT
ENSG00000112379	797.32	2.176	8.46E-17	1.26E-12	ARFGEF3
ENSG00000198074	137.81	-3.105	1.00E-16	1.49E-12	AKR1B10
ENSG00000173918	216.50	-2.428	1.33E-16	1.98E-12	C1QTNF1

ENSG00000184185	117.78	-3.320	1.43E-16	2.13E-12	KCNJ12
ENSG00000185053	727.26	1.824	1.63E-16	2.42E-12	SGCZ
ENSG00000135549	1384.19	-1.808	2.62E-16	3.90E-12	PKIB
ENSG00000183421	591.80	-1.976	3.03E-16	4.50E-12	RIPK4
ENSG00000182054	1250.47	1.674	3.07E-16	4.57E-12	IDH2
ENSG00000110723	120.69	3.194	3.38E-16	5.02E-12	EXPH5
ENSG00000167191	400.83	2.042	4.04E-16	6.01E-12	GPRC5B
ENSG00000148154	13360.76	1.521	4.23E-16	6.29E-12	UGCG
ENSG00000120694	17733.51	-1.535	4.68E-16	6.97E-12	HSPH1
ENSG00000183128	93.97	-3.543	5.20E-16	7.73E-12	CALHM3
ENSG00000116132	1221.99	-1.915	5.55E-16	8.26E-12	PRRX1
ENSG00000250312	152.95	2.742	7.12E-16	1.06E-11	ZNF718
ENSG00000182111	377.50	2.200	7.58E-16	1.13E-11	ZNF716
ENSG00000131016	14208.65	2.089	9.17E-16	1.36E-11	AKAP12
ENSG00000105472	285.10	2.174	1.37E-15	2.04E-11	CLEC11A
ENSG00000109743	203.41	2.439	1.76E-15	2.62E-11	BST1
ENSG00000106078	126.59	3.025	1.77E-15	2.64E-11	COBL
ENSG00000125810	100.04	-3.378	1.89E-15	2.81E-11	CD93
ENSG00000148053	123.58	-3.264	2.41E-15	3.58E-11	NTRK2
ENSG00000143322	13084.84	1.775	2.41E-15	3.58E-11	ABL2
ENSG00000184524	155.21	-2.673	3.31E-15	4.93E-11	CEND1
ENSG00000165959	421.16	1.921	3.53E-15	5.25E-11	CLMN
ENSG00000165509	103.92	3.326	3.83E-15	5.70E-11	MAGEC3
ENSG00000142949	41041.94	1.838	5.97E-15	8.87E-11	PTPRF
ENSG00000178752	187.15	2.420	6.00E-15	8.92E-11	ERFE
ENSG00000078018	302.81	-3.063	6.19E-15	9.21E-11	MAP2
ENSG00000159216	1289.49	1.259	1.97E-06	2.93E-02	RUNX1
ENSG00000115423	102.18	3.344	8.15E-15	1.21E-10	DNAH6
ENSG00000162614	513.17	1.869	8.75E-15	1.30E-10	NEXN
ENSG00000142279	223.93	2.368	8.85E-15	1.32E-10	WTIP
ENSG00000148848	269.66	-2.190	1.18E-14	1.76E-10	ADAM12
ENSG00000197444	170.59	2.484	1.39E-14	2.06E-10	OGDHL
ENSG00000169908	15155.74	1.448	1.69E-14	2.51E-10	TM4SF1
ENSG00000128606	841.58	-2.054	1.70E-14	2.52E-10	LRRC17
ENSG00000109511	401.90	-1.947	1.73E-14	2.57E-10	ANXA10
ENSG00000176658	293.19	2.056	2.03E-14	3.02E-10	MYO1D
ENSG00000086061	13689.08	-1.425	2.05E-14	3.04E-10	DNAJA1
ENSG00000141526	895.84	-1.774	2.74E-14	4.07E-10	SLC16A3
ENSG00000165449	198.15	2.375	3.03E-14	4.50E-10	SLC16A9
ENSG00000105509	80.77	-6.912	3.16E-14	4.70E-10	HAS1
ENSG00000115525	704.32	-1.711	3.27E-14	4.87E-10	ST3GAL5

ENSG00000071575	1349.34	-1.663	3.34E-14	4.97E-10	TRIB2
ENSG00000128342	3784.57	1.453	3.36E-14	5.00E-10	LIF
ENSG00000255346	227.81	-2.294	4.11E-14	6.12E-10	NOX5
ENSG00000124785	355.16	2.045	4.29E-14	6.38E-10	NRN1
ENSG00000169439	835.24	-2.038	4.69E-14	6.98E-10	SDC2
ENSG00000115461	65.93	-3.903	4.72E-14	7.02E-10	IGFBP5
ENSG00000157551	91.44	-3.502	5.49E-14	8.17E-10	KCNJ15
ENSG00000196502	87.68	-3.519	5.70E-14	8.48E-10	SULT1A1
ENSG00000081138	251.57	-2.418	6.09E-14	9.06E-10	CDH7
ENSG00000047617	135.23	3.016	6.67E-14	9.92E-10	ANO2
ENSG00000138166	3630.58	1.444	6.71E-14	9.98E-10	DUSP5
ENSG00000130303	99.35	-3.347	8.55E-14	1.27E-09	BST2
ENSG00000182752	393.54	-1.919	8.87E-14	1.32E-09	PAPPA
ENSG00000175197	1841.59	1.797	1.00E-13	1.49E-09	DDIT3
ENSG00000109321	2520.09	-1.879	1.02E-13	1.51E-09	AREG
ENSG00000185274	81.53	3.529	1.03E-13	1.54E-09	GALNT17
ENSG00000188015	826.42	-1.633	1.36E-13	2.03E-09	S100A3
ENSG00000011201	183.40	2.304	1.43E-13	2.13E-09	ANOS1
ENSG00000164099	835.31	1.592	1.47E-13	2.19E-09	PRSS12
ENSG00000178031	3630.67	1.766	1.50E-13	2.23E-09	ADAMTSL1
ENSG00000080031	347.04	1.892	1.57E-13	2.33E-09	PTPRH
ENSG00000162631	528.49	1.785	1.67E-13	2.49E-09	NTNG1
ENSG00000134532	1133.83	-1.515	1.81E-13	2.70E-09	SOX5
ENSG00000134762	436.14	2.020	1.89E-13	2.81E-09	DSC3
ENSG00000112773	688.85	-1.731	1.99E-13	2.96E-09	TENT5A
ENSG00000112851	18465.08	1.370	2.32E-13	3.44E-09	ERBIN
ENSG00000067445	104.52	-2.896	2.39E-13	3.55E-09	TRO
ENSG00000185905	175.41	-2.315	2.44E-13	3.64E-09	C16orf54
ENSG00000173597	128.22	-2.549	2.53E-13	3.76E-09	SULT1B1
ENSG00000054654	756.73	1.973	2.61E-13	3.88E-09	SYNE2
ENSG00000168874	138.55	-2.465	2.62E-13	3.90E-09	ATOH8
ENSG00000162676	123.81	2.722	2.84E-13	4.23E-09	GFI1
ENSG00000196110	746.69	1.592	3.01E-13	4.48E-09	ZNF699
ENSG00000169126	641.04	1.636	3.03E-13	4.51E-09	ARMC4
ENSG00000105376	762.77	1.635	3.11E-13	4.63E-09	ICAM5
ENSG00000163694	259.66	-2.014	3.48E-13	5.17E-09	RBM47
ENSG00000204442	886.35	-1.672	4.78E-13	7.11E-09	FAM155A
ENSG00000174343	83.69	-3.730	4.86E-13	7.23E-09	CHRNA9
ENSG00000092969	339.53	-1.935	4.93E-13	7.34E-09	TGFB2
ENSG00000140044	490.95	1.721	5.25E-13	7.81E-09	JDP2
ENSG00000152377	1317.01	-1.506	5.53E-13	8.23E-09	SPOCK1

ENSG00000101188	209.76	-2.597	5.62E-13	8.35E-09	NTSR1
ENSG00000176595	93.13	3.169	5.68E-13	8.46E-09	KBTBD11
ENSG00000163412	329.56	1.872	5.97E-13	8.88E-09	EIF4E3
ENSG00000100146	645.64	-1.879	6.01E-13	8.94E-09	SOX10
ENSG00000170689	495.18	1.805	6.68E-13	9.94E-09	HOXB9
ENSG00000259207	1484.04	-1.536	6.78E-13	1.01E-08	ITGB3
ENSG00000064042	6671.63	1.355	7.37E-13	1.10E-08	LIMCH1
ENSG00000174136	2448.87	1.470	7.45E-13	1.11E-08	RGMB
ENSG00000138131	4964.13	1.708	8.06E-13	1.20E-08	LOXL4
ENSG00000151892	72.16	3.657	8.53E-13	1.27E-08	GFRA1
ENSG00000033327	468.43	2.042	1.01E-12	1.50E-08	GAB2
ENSG00000121413	643.60	1.597	1.14E-12	1.69E-08	ZSCAN18
ENSG00000184489	63.11	4.132	1.16E-12	1.72E-08	PTP4A3
ENSG00000163884	102.81	3.172	1.28E-12	1.91E-08	KLF15
ENSG00000196754	1947.40	-1.503	1.33E-12	1.99E-08	S100A2
ENSG00000023608	10356.72	1.714	1.35E-12	2.00E-08	SNAPC1
ENSG00000119711	614.59	1.599	1.50E-12	2.23E-08	ALDH6A1
ENSG00000087074	4324.70	1.552	1.76E-12	2.61E-08	PPP1R15A
ENSG00000136010	2544.52	1.641	1.90E-12	2.83E-08	ALDH1L2
ENSG00000137193	1272.12	1.681	1.94E-12	2.89E-08	PIM1
ENSG00000066056	785.83	1.742	1.99E-12	2.95E-08	TIE1
ENSG00000183691	714.89	2.108	1.99E-12	2.96E-08	NOG
ENSG00000107099	55.31	-4.731	2.09E-12	3.12E-08	DOCK8
ENSG00000163092	75.58	3.360	2.13E-12	3.17E-08	XIRP2
ENSG00000125266	1177.48	-1.526	2.63E-12	3.92E-08	EFNB2
ENSG00000164983	1359.16	1.444	2.68E-12	3.98E-08	TMEM65
ENSG00000119950	1698.73	1.396	3.18E-12	4.74E-08	MXI1
ENSG00000198914	98.60	2.780	4.59E-12	6.82E-08	POU3F3
ENSG00000139626	222.58	-1.962	4.65E-12	6.92E-08	ITGB7
ENSG00000113303	98.64	2.793	4.91E-12	7.30E-08	BTNL8
ENSG00000196876	376.45	1.737	5.06E-12	7.53E-08	SCN8A
ENSG00000044524	339.32	1.771	5.19E-12	7.71E-08	EPHA3
ENSG00000111879	302.80	1.900	5.66E-12	8.42E-08	FAM184A
ENSG00000163762	559.55	1.599	5.89E-12	8.76E-08	TM4SF18
ENSG00000163032	257.63	-2.031	7.58E-12	1.13E-07	VSNL1
ENSG00000176293	356.65	1.761	8.24E-12	1.23E-07	ZNF135
ENSG00000160321	201.55	2.586	9.31E-12	1.39E-07	ZNF208
ENSG00000116741	762.68	-1.887	1.07E-11	1.59E-07	RGS2
ENSG00000151062	104.12	2.873	1.08E-11	1.61E-07	CACNA2D4
ENSG00000100889	2335.89	1.409	1.08E-11	1.61E-07	PCK2
ENSG00000134755	293.48	1.826	1.14E-11	1.70E-07	DSC2

ENSG00000008256	2737.71	1.328	1.20E-11	1.78E-07	CYTH3
ENSG00000188488	217.48	-2.142	1.28E-11	1.90E-07	SERPINA5
ENSG00000134954	6548.66	1.171	1.11E-07	1.66E-03	ETS1
ENSG00000123358	546.61	1.598	1.32E-11	1.97E-07	NR4A1
ENSG00000113504	559.55	1.667	1.35E-11	2.01E-07	SLC12A7
ENSG00000164683	179.76	-2.574	1.63E-11	2.42E-07	HEY1
ENSG00000111674	3017.24	1.372	1.63E-11	2.42E-07	ENO2
ENSG00000133687	230.02	-1.962	1.65E-11	2.45E-07	TMTC1
ENSG00000150054	506.29	1.740	1.72E-11	2.55E-07	MPP7
ENSG00000142619	227.68	-1.953	1.86E-11	2.76E-07	PADI3
ENSG00000099860	967.27	1.413	1.90E-11	2.83E-07	GADD45B
ENSG00000183873	67.96	3.447	1.91E-11	2.84E-07	SCN5A
ENSG00000009694	152.02	2.231	2.18E-11	3.24E-07	TENM1
ENSG00000070540	1131.95	1.611	2.29E-11	3.41E-07	WIPI1
ENSG00000166833	324.95	2.259	2.64E-11	3.93E-07	NAV2
ENSG00000205426	26980.46	1.316	2.73E-11	4.07E-07	KRT81
ENSG00000179242	516.19	2.196	3.01E-11	4.48E-07	CDH4
ENSG00000168209	11762.58	1.801	3.85E-11	5.73E-07	DDIT4
ENSG00000117152	1126.46	-1.389	4.29E-11	6.39E-07	RGS4
ENSG00000198797	54.62	3.947	4.30E-11	6.40E-07	BRINP2
ENSG00000125798	110.90	3.204	4.49E-11	6.68E-07	FOXA2
ENSG00000163531	372.23	1.752	4.60E-11	6.84E-07	NFASC
ENSG00000134986	1066.59	-1.881	4.73E-11	7.04E-07	NREP
ENSG00000149571	280.55	1.780	4.74E-11	7.05E-07	KIRREL3
ENSG00000172020	161.77	-2.466	4.89E-11	7.27E-07	GAP43
ENSG00000186469	483.48	-1.770	5.33E-11	7.93E-07	GNG2
ENSG00000136237	768.00	1.426	5.35E-11	7.95E-07	RAPGEF5
ENSG00000187244	530.91	1.622	6.22E-11	9.26E-07	BCAM
ENSG00000140481	90.53	-2.853	6.50E-11	9.67E-07	CCDC33
ENSG00000164197	255.67	1.795	7.61E-11	1.13E-06	RNF180
ENSG00000082397	2303.82	1.333	7.70E-11	1.15E-06	EPB41L3
ENSG00000168461	1054.08	-1.370	8.13E-11	1.21E-06	RAB31
ENSG00000188467	265.96	1.895	9.21E-11	1.37E-06	SLC24A5
ENSG00000152137	1152.70	-1.349	1.09E-10	1.63E-06	HSPB8
ENSG00000159339	71.71	-7.373	1.13E-10	1.68E-06	PADI4
ENSG00000126860	388.03	1.653	1.19E-10	1.77E-06	EVI2A
ENSG00000139269	129.74	2.953	1.20E-10	1.78E-06	INHBE
ENSG00000166106	413.68	-1.678	1.29E-10	1.92E-06	ADAMTS15
ENSG00000266338	2207.75	1.340	1.29E-10	1.93E-06	NBPF15
ENSG00000174405	1679.30	-1.503	1.47E-10	2.19E-06	LIG4
ENSG00000110665	41.30	-4.534	1.64E-10	2.44E-06	C11orf21

ENSG00000100784	524.74	1.517	1.76E-10	2.62E-06	RPS6KA5
ENSG00000153993	1558.08	1.335	1.79E-10	2.66E-06	SEMA3D
ENSG00000112769	10806.00	1.406	1.81E-10	2.70E-06	LAMA4
ENSG00000112972	5762.65	1.586	1.97E-10	2.94E-06	HMGCS1
ENSG00000137745	48.08	-4.049	2.10E-10	3.12E-06	MMP13
ENSG00000135362	192.88	-1.883	2.40E-10	3.57E-06	PRR5L
ENSG00000006042	154.10	2.092	2.57E-10	3.83E-06	TMEM98
ENSG00000117461	563.54	-1.431	2.58E-10	3.83E-06	PIK3R3
ENSG00000130635	896.43	-1.945	2.85E-10	4.25E-06	COL5A1
ENSG00000166402	338.23	1.647	2.86E-10	4.25E-06	TUB
ENSG00000067798	1968.96	1.248	3.22E-10	4.80E-06	NAV3
ENSG00000164283	3324.95	1.365	3.27E-10	4.86E-06	ESM1
ENSG00000143847	248.34	1.756	3.43E-10	5.11E-06	PPFIA4
ENSG00000125657	1049.26	-1.320	4.00E-10	5.95E-06	TNFSF9
ENSG00000106484	185.37	-2.164	4.05E-10	6.03E-06	MEST
ENSG00000129422	755.08	1.399	4.15E-10	6.18E-06	MTUS1
ENSG00000108821	253.29	2.328	4.20E-10	6.24E-06	COL1A1
ENSG00000079263	343.32	-1.578	4.50E-10	6.70E-06	SP140
ENSG00000138623	8525.02	1.213	4.64E-10	6.90E-06	SEMA7A
ENSG00000075651	896.96	1.319	4.71E-10	7.01E-06	PLD1
ENSG00000116667	1095.47	1.288	4.75E-10	7.07E-06	C1orf21
ENSG00000069020	472.52	1.815	5.22E-10	7.77E-06	MAST4
ENSG00000265190	67.08	-3.033	5.42E-10	8.06E-06	ANXA8
ENSG00000144749	1351.71	1.532	5.45E-10	8.11E-06	LRIG1
ENSG00000167972	98.60	2.421	6.83E-10	1.02E-05	ABCA3
ENSG00000155629	369.65	1.652	6.87E-10	1.02E-05	PIK3AP1
ENSG00000178038	331.07	1.621	7.52E-10	1.12E-05	ALS2CL
ENSG00000118777	718.80	-1.336	7.77E-10	1.16E-05	ABCG2
ENSG00000151012	3761.13	1.349	9.43E-10	1.40E-05	SLC7A11
ENSG00000148926	947.58	1.413	9.47E-10	1.41E-05	ADM
ENSG00000018236	251.48	1.723	1.06E-09	1.57E-05	CNTN1
ENSG00000136531	339.29	-1.733	1.09E-09	1.62E-05	SCN2A
ENSG00000173801	148.34	-2.032	1.19E-09	1.77E-05	JUP
ENSG00000130751	290.68	1.599	1.21E-09	1.80E-05	NPAS1
ENSG00000137809	91.85	-2.547	1.34E-09	2.00E-05	ITGA11
ENSG00000168398	38.61	-4.623	1.34E-09	2.00E-05	BDKRB2
ENSG00000205213	526.43	-1.439	1.41E-09	2.09E-05	LGR4
ENSG00000085117	114.80	-2.152	1.45E-09	2.15E-05	CD82
ENSG00000151490	964.67	-1.567	1.45E-09	2.15E-05	PTPRO
ENSG00000254585	42.56	4.634	1.46E-09	2.17E-05	MAGEL2
ENSG00000137571	71.96	2.910	1.53E-09	2.27E-05	SLCO5A1

ENSG00000197102	25978.21	1.953	1.53E-09	2.28E-05	DYNC1H1
ENSG00000099937	70.80	-2.773	1.56E-09	2.32E-05	SERPIND1
ENSG00000177504	59.29	-3.233	1.62E-09	2.41E-05	VCX2
ENSG00000171049	90.09	-8.671	1.68E-09	2.50E-05	FPR2
ENSG00000170412	65.83	-2.759	1.89E-09	2.80E-05	GPRC5C
ENSG00000182795	82.55	2.624	1.92E-09	2.85E-05	C1orf116
ENSG00000147041	396.91	-1.460	2.00E-09	2.97E-05	SYTL5
ENSG00000136250	180.02	1.852	2.03E-09	3.01E-05	AOAH
ENSG00000160145	118.46	-2.089	2.04E-09	3.04E-05	KALRN
ENSG00000171105	614.49	1.640	2.13E-09	3.17E-05	INSR
ENSG00000119121	108.90	-2.201	2.19E-09	3.26E-05	TRPM6
ENSG00000165171	661.79	1.321	2.24E-09	3.33E-05	METTL27
ENSG00000185818	748.25	1.378	2.40E-09	3.57E-05	NAT8L
ENSG00000146592	91.86	2.405	2.53E-09	3.77E-05	CREB5
ENSG00000205777	109.36	2.246	2.88E-09	4.28E-05	GAGE1
ENSG00000163975	5347.92	1.211	2.94E-09	4.38E-05	MELTF
ENSG00000130052	527.97	1.389	3.07E-09	4.57E-05	STARD8
ENSG00000157617	54.25	-3.023	3.14E-09	4.67E-05	C2CD2
ENSG00000185432	148.37	-2.182	3.25E-09	4.83E-05	METTL7A
ENSG00000169122	44.88	-3.396	3.39E-09	5.04E-05	FAM110B
ENSG00000211445	436.50	-1.454	3.40E-09	5.06E-05	GPX3
ENSG00000154678	14129.63	1.159	3.46E-09	5.15E-05	PDE1C
ENSG00000125398	3169.90	1.643	3.49E-09	5.19E-05	SOX9
ENSG00000144619	92.23	2.568	3.51E-09	5.22E-05	CNTN4
ENSG00000120738	132.40	2.137	3.72E-09	5.53E-05	EGR1
ENSG00000147394	245.04	1.901	4.10E-09	6.09E-05	ZNF185
ENSG00000023445	613.25	1.339	4.15E-09	6.18E-05	BIRC3
ENSG00000164070	1157.16	-1.206	4.21E-09	6.27E-05	HSPA4L
ENSG00000185567	2119.01	1.414	4.29E-09	6.37E-05	AHNAK2
ENSG00000124107	42.72	-3.860	4.43E-09	6.58E-05	SLPI
ENSG00000154274	173.89	-1.882	4.53E-09	6.74E-05	C4orf19
ENSG00000111696	5758.44	1.371	4.60E-09	6.84E-05	NT5DC3
ENSG00000259753	1059.36	-1.338	5.14E-09	7.64E-05	AC068234.1
ENSG00000197565	122.95	-2.043	5.26E-09	7.82E-05	COL4A6
ENSG00000080573	47.39	-3.345	5.70E-09	8.48E-05	COL5A3
ENSG00000163132	2877.28	-1.132	5.88E-09	8.74E-05	MSX1
ENSG00000150551	1115.97	-1.225	6.07E-09	9.03E-05	LYPD1
ENSG00000052802	5336.54	1.370	6.40E-09	9.52E-05	MSMO1
ENSG00000134955	1003.95	-1.238	6.44E-09	9.58E-05	SLC37A2
ENSG00000198795	85.65	2.417	6.55E-09	9.74E-05	ZNF521
ENSG00000156804	590.24	1.388	6.57E-09	9.77E-05	FBXO32

ENSG00000144802	829.92	1.252	6.62E-09	9.84E-05	NFKBIZ
ENSG00000170381	243.71	1.614	7.00E-09	1.04E-04	SEMA3E
ENSG00000111490	77.75	-2.484	7.13E-09	1.06E-04	TBC1D30
ENSG00000123384	3378.40	1.552	7.51E-09	1.12E-04	LRP1
ENSG00000185716	3430.15	1.127	8.09E-09	1.20E-04	MOSMO
ENSG00000205309	194.89	1.730	8.48E-09	1.26E-04	NT5M
ENSG00000101752	8759.79	1.084	8.49E-09	1.26E-04	MIB1
ENSG00000128165	165.99	1.804	8.55E-09	1.27E-04	ADM2
ENSG00000065361	188.90	1.753	8.89E-09	1.32E-04	ERBB3
ENSG00000143333	999.63	1.217	9.32E-09	1.39E-04	RGS16
ENSG00000155324	362.58	1.519	9.74E-09	1.45E-04	GRAMD2B
ENSG00000159403	121.14	2.051	9.92E-09	1.48E-04	C1R
ENSG00000130762	60.32	-2.850	1.01E-08	1.50E-04	ARHGEF16
ENSG00000163421	52.30	3.193	1.03E-08	1.53E-04	PROK2
ENSG00000178922	2637.84	1.203	1.05E-08	1.56E-04	HYI
ENSG00000101695	450.64	1.374	1.08E-08	1.61E-04	RNF125
ENSG00000140015	56.68	3.071	1.10E-08	1.64E-04	KCNH5
ENSG00000181577	59.70	2.884	1.17E-08	1.75E-04	C6orf223
ENSG00000205403	231.72	-2.249	1.17E-08	1.75E-04	CFI
ENSG00000112414	4052.55	1.141	1.24E-08	1.85E-04	ADGRG6
ENSG00000182168	38.88	-3.791	1.30E-08	1.93E-04	UNC5C
ENSG00000143473	156.11	1.883	1.34E-08	2.00E-04	KCNH1
ENSG00000244694	300.84	-1.498	1.43E-08	2.12E-04	PTCHD4
ENSG00000130881	257.54	1.656	1.46E-08	2.17E-04	LRP3
ENSG00000146966	2363.91	1.331	1.60E-08	2.38E-04	DENND2A
ENSG00000182272	755.66	1.227	1.60E-08	2.38E-04	B4GALNT4
ENSG00000186480	7870.44	1.262	1.63E-08	2.42E-04	INSIG1
ENSG00000122966	3251.18	1.578	1.65E-08	2.45E-04	CIT
ENSG00000166073	2825.51	1.206	1.72E-08	2.55E-04	GPR176
ENSG00000100292	924.69	-1.191	1.75E-08	2.60E-04	HMOX1
ENSG00000167995	15140.37	-1.133	1.83E-08	2.72E-04	BEST1
ENSG00000169169	134.36	2.307	1.84E-08	2.73E-04	CPT1C
ENSG00000129353	1106.11	1.305	1.88E-08	2.79E-04	SLC44A2
ENSG00000049323	609.93	-1.252	1.93E-08	2.88E-04	LTBP1
ENSG00000169306	957.14	1.200	1.98E-08	2.94E-04	IL1RAPL1
ENSG00000186297	407.49	1.411	1.98E-08	2.94E-04	GABRA5
ENSG00000135114	362.91	-1.529	2.00E-08	2.97E-04	OASL
ENSG00000153233	125.00	2.121	2.13E-08	3.17E-04	PTPRR
ENSG00000175344	130.75	1.906	2.16E-08	3.21E-04	CHRNA7
ENSG00000166396	3779.16	1.189	2.22E-08	3.31E-04	SERPINB7
ENSG00000065325	195.96	-1.763	2.23E-08	3.32E-04	GLP2R

ENSG00000162723	98.57	2.156	2.39E-08	3.55E-04	SLAMF9
ENSG00000124762	7222.69	-1.159	2.39E-08	3.55E-04	CDKN1A
ENSG00000171608	1101.31	1.197	2.75E-08	4.09E-04	PIK3CD
ENSG00000143387	158.80	-2.215	2.75E-08	4.09E-04	CTSK
ENSG00000127252	53.11	2.939	2.80E-08	4.17E-04	HRASLS
ENSG00000138669	154.73	1.777	3.00E-08	4.46E-04	PRKG2
ENSG00000107295	1723.39	1.192	3.12E-08	4.63E-04	SH3GL2
ENSG00000143603	83.70	-2.402	3.21E-08	4.77E-04	KCNN3
ENSG00000107719	125.18	1.924	3.24E-08	4.82E-04	PALD1
ENSG00000152217	270.88	-1.491	3.27E-08	4.86E-04	SETBP1
ENSG00000133067	61.22	-2.832	3.45E-08	5.13E-04	LGR6
ENSG00000135472	101.14	-2.142	3.45E-08	5.13E-04	FAIM2
ENSG00000185070	604.39	-1.245	3.57E-08	5.31E-04	FLRT2
ENSG00000152223	2852.96	1.249	3.62E-08	5.38E-04	EPG5
ENSG00000072274	20115.41	-1.226	3.84E-08	5.72E-04	TFRC
ENSG00000081665	294.41	1.457	4.21E-08	6.26E-04	ZNF506
ENSG00000205269	353.87	1.400	4.27E-08	6.36E-04	TMEM170B
ENSG00000165272	104.86	-2.083	4.37E-08	6.51E-04	AQP3
ENSG00000101255	11658.26	1.031	4.51E-08	6.71E-04	TRIB3
ENSG00000110987	324.76	1.397	4.57E-08	6.79E-04	BCL7A
ENSG00000158859	79.67	-2.364	4.64E-08	6.91E-04	ADAMTS4
ENSG00000124942	39836.39	1.747	4.83E-08	7.18E-04	AHNAK
ENSG00000111266	906.46	1.156	4.90E-08	7.29E-04	DUSP16
ENSG00000185022	1263.00	1.152	2.17E-07	3.23E-03	MAFF
ENSG00000046774	11698.97	-1.128	5.09E-08	7.57E-04	MAGEC2
ENSG00000169554	458.54	1.324	5.13E-08	7.63E-04	ZEB2
ENSG00000221955	130.20	-1.953	5.32E-08	7.92E-04	SLC12A8
ENSG00000065618	378.95	-1.610	5.34E-08	7.95E-04	COL17A1
ENSG00000255690	47.42	-2.894	6.09E-08	9.06E-04	TRIL
ENSG00000104369	803.76	1.172	6.42E-08	9.54E-04	JPH1
ENSG00000164741	7321.56	1.116	6.55E-08	9.75E-04	DLC1
ENSG00000171298	1424.72	1.492	6.58E-08	9.79E-04	GAA
ENSG00000265118	182.87	1.623	6.59E-08	9.80E-04	AC134669.1
ENSG00000143494	158.31	1.743	6.63E-08	9.86E-04	VASH2
ENSG00000136161	189.19	-1.648	6.75E-08	1.00E-03	RCBTB2
ENSG00000170537	716.05	1.177	6.79E-08	1.01E-03	TMC7
ENSG00000130508	952.52	1.217	8.34E-08	1.24E-03	PXDN
ENSG00000106003	405.04	-1.345	8.69E-08	1.29E-03	LFNG
ENSG00000168994	1478.95	1.148	8.70E-08	1.29E-03	PXDC1
ENSG00000130433	99.13	2.041	8.83E-08	1.31E-03	CACNG6
ENSG00000079931	284.01	1.425	8.93E-08	1.33E-03	MOXD1

ENSG00000137501	1597.37	1.094	9.07E-08	1.35E-03	SYTL2
ENSG00000105767	952.39	1.125	9.12E-08	1.36E-03	CADM4
ENSG00000142173	8578.53	1.196	9.50E-08	1.41E-03	COL6A2
ENSG00000103811	540.50	1.344	9.54E-08	1.42E-03	CTSH
ENSG00000163686	592.83	-1.221	9.91E-08	1.47E-03	ABHD6
ENSG00000148120	1263.95	-1.118	1.02E-07	1.51E-03	C9orf3
ENSG00000145040	400.25	-1.295	1.02E-07	1.52E-03	UCN2
ENSG00000155974	281.54	-1.527	1.05E-07	1.57E-03	GRIP1
ENSG00000154813	2038.04	-1.136	1.07E-07	1.59E-03	DPH3
ENSG00000143127	856.60	1.165	1.07E-07	1.59E-03	ITGA10
ENSG00000116584	14785.48	1.099	1.21E-06	1.79E-02	ARHGEF2
ENSG00000114126	1439.38	1.077	1.43E-07	2.12E-03	TFDP2
ENSG00000049759	7944.29	1.499	1.30E-07	1.94E-03	NEDD4L
ENSG00000136717	289.55	-1.498	1.31E-07	1.95E-03	BIN1
ENSG00000196350	101.26	2.009	1.33E-07	1.98E-03	ZNF729
ENSG00000133639	3530.96	1.072	4.92E-08	7.32E-04	BTG1
ENSG00000031081	281.68	-1.420	1.35E-07	2.00E-03	ARHGAP31
ENSG00000135048	2364.85	-1.040	1.40E-07	2.08E-03	CEMIP2
ENSG00000119900	6114.42	1.005	1.34E-07	1.99E-03	OGFRL1
ENSG00000085662	11713.08	-1.087	1.44E-07	2.14E-03	AKR1B1
ENSG00000172428	1219.74	-1.169	1.46E-07	2.17E-03	COPS9
ENSG00000183780	834.08	1.276	1.53E-07	2.28E-03	SLC35F3
ENSG00000169129	42.97	-3.148	1.59E-07	2.36E-03	AFAP1L2
ENSG00000124610	453.06	-1.260	1.67E-07	2.48E-03	HIST1H1A
ENSG00000100473	511.57	1.224	1.67E-07	2.48E-03	COCH
ENSG00000094631	1479.15	1.126	1.80E-07	2.68E-03	HDAC6
ENSG00000116717	2491.36	1.019	1.83E-07	2.72E-03	GADD45A
ENSG00000118503	1195.84	-1.090	1.86E-07	2.76E-03	TNFAIP3
ENSG00000101084	5334.70	-1.027	1.90E-07	2.82E-03	RAB5IF
ENSG00000131242	96.12	2.086	1.97E-07	2.93E-03	RAB11FIP4
ENSG00000115828	553.88	-1.551	2.14E-07	3.19E-03	QPCT
ENSG00000169213	2555.67	1.325	2.17E-07	3.23E-03	RAB3B
ENSG00000076513	3050.29	1.033	2.17E-07	3.23E-03	ANKRD13A
ENSG00000163453	1643.95	-1.392	1.15E-07	1.71E-03	IGFBP7
ENSG00000167996	26007.78	-1.104	2.26E-07	3.36E-03	FTH1
ENSG00000172432	2028.97	1.035	2.29E-07	3.40E-03	GTPBP2
ENSG00000196586	1361.16	1.214	2.38E-07	3.54E-03	MYO6
ENSG00000239713	2675.81	-1.223	2.42E-07	3.59E-03	APOBEC3G
ENSG00000179873	283.93	1.548	2.42E-07	3.60E-03	NLRP11
ENSG00000172828	139.77	1.701	2.45E-07	3.65E-03	CES3
ENSG00000144218	41.52	3.324	2.66E-07	3.96E-03	AFF3

ENSG00000170006	3333.70	1.075	2.68E-07	3.99E-03	TMEM154
ENSG00000142552	111.17	1.879	2.77E-07	4.12E-03	RCN3
ENSG00000251537	1019.72	-1.095	2.79E-07	4.15E-03	AC005324.3
ENSG00000181649	1026.85	-1.089	2.90E-07	4.31E-03	PHLDA2
ENSG00000223802	802.95	1.259	2.95E-07	4.39E-03	CERS1
ENSG00000106624	2697.82	1.438	3.04E-07	4.52E-03	AEBP1
ENSG00000167601	46882.64	1.222	3.04E-07	4.53E-03	AXL
ENSG00000071991	30.86	4.355	3.06E-07	4.55E-03	CDH19
ENSG00000132182	1051.37	1.352	3.17E-07	4.71E-03	NUP210
ENSG00000116299	60.47	-2.549	3.35E-07	4.98E-03	KIAA1324
ENSG00000168477	84.14	-2.283	3.38E-07	5.03E-03	TNXB
ENSG00000185507	139.07	-1.712	3.41E-07	5.07E-03	IRF7
ENSG00000165716	308.32	1.444	3.49E-07	5.19E-03	FAM69B
ENSG00000169252	663.21	1.174	3.78E-07	5.62E-03	ADRB2
ENSG00000188177	375.66	1.462	3.86E-07	5.75E-03	ZC3H6
ENSG00000182636	1664.85	1.015	3.87E-07	5.75E-03	NDN
ENSG00000087253	2118.26	-1.011	3.89E-07	5.78E-03	LPCAT2
ENSG00000100031	63.29	-2.277	3.94E-07	5.86E-03	GGT1
ENSG00000151067	64.87	2.406	4.06E-07	6.03E-03	CACNA1C
ENSG00000100814	2168.72	1.179	4.11E-07	6.12E-03	CCNB1IP1
ENSG00000165821	75.61	2.143	4.16E-07	6.18E-03	SALL2
ENSG00000260170	554.40	1.236	4.19E-07	6.23E-03	AC090527.2
ENSG00000180287	29.52	4.307	4.19E-07	6.23E-03	PLD5
ENSG00000104856	1009.72	1.101	4.41E-07	6.56E-03	RELB
ENSG00000147676	592.33	1.219	4.47E-07	6.65E-03	MAL2
ENSG00000069431	144.23	1.679	4.61E-07	6.86E-03	ABCC9
ENSG00000149150	1134.37	1.039	4.62E-07	6.87E-03	SLC43A1
ENSG00000143816	109.92	-2.217	4.91E-07	7.31E-03	WNT9A
ENSG00000091972	47.57	7.397	5.06E-07	7.53E-03	CD200
ENSG00000157303	77.27	2.113	5.19E-07	7.72E-03	SUSD3
ENSG00000103942	847.82	1.138	5.19E-07	7.72E-03	HOMER2
ENSG00000145536	115.24	-1.778	5.33E-07	7.92E-03	ADAMTS16
ENSG00000140450	217.35	1.467	5.34E-07	7.95E-03	ARRDC4
ENSG00000002587	355.85	1.282	5.46E-07	8.11E-03	HS3ST1
ENSG00000091622	370.52	1.471	5.48E-07	8.15E-03	PITPNM3
ENSG00000188277	228.27	-1.589	5.52E-07	8.21E-03	C15orf62
ENSG00000132963	7802.92	-1.052	5.62E-07	8.36E-03	POMP
ENSG00000085871	353.96	-1.263	5.80E-07	8.63E-03	MGST2
ENSG00000141682	6700.78	1.225	5.91E-07	8.80E-03	PMAIP1
ENSG00000074771	31.46	5.809	5.98E-07	8.89E-03	NOX3
ENSG00000113739	1526.50	1.050	6.00E-07	8.93E-03	STC2

ENSG00000069667	350.41	1.306	6.02E-07	8.95E-03	RORA
ENSG00000133863	210.26	1.513	6.14E-07	9.14E-03	TEX15
ENSG00000149489	359.15	-1.228	6.52E-07	9.70E-03	ROM1
ENSG00000198211	10582.54	-1.051	6.55E-07	9.74E-03	AC092143.1
ENSG00000148803	409.12	1.504	6.57E-07	9.77E-03	FUOM
ENSG00000176406	97.37	1.944	6.86E-07	1.02E-02	RIMS2
ENSG00000150782	902.57	1.154	6.92E-07	1.03E-02	IL18
ENSG00000064201	40.09	-2.929	7.21E-07	1.07E-02	TSPAN32
ENSG00000258947	10787.04	-1.052	7.61E-07	1.13E-02	TUBB3
ENSG00000198198	4207.92	1.092	7.64E-07	1.14E-02	SZT2
ENSG00000136114	120.11	1.763	7.65E-07	1.14E-02	THSD1
ENSG00000197019	603.17	-1.110	7.98E-07	1.19E-02	SERTAD1
ENSG00000003147	466.26	1.310	8.06E-07	1.20E-02	ICA1
ENSG00000198574	164.54	-1.508	8.06E-07	1.20E-02	SH2D1B
ENSG00000160867	285.57	1.286	8.62E-07	1.28E-02	FGFR4
ENSG00000133315	1313.37	1.018	8.63E-07	1.28E-02	MACROD1
ENSG00000152270	348.93	1.385	8.75E-07	1.30E-02	PDE3B
ENSG00000158321	151.53	1.599	8.76E-07	1.30E-02	AUTS2
ENSG00000182583	34.42	-3.375	8.77E-07	1.31E-02	VCX
ENSG00000021645	40.54	3.182	9.13E-07	1.36E-02	NRXN3
ENSG00000140600	94.28	1.989	9.18E-07	1.37E-02	SH3GL3
ENSG00000148408	30.50	5.769	9.37E-07	1.39E-02	CACNA1B
ENSG00000111799	1596.66	1.465	9.62E-07	1.43E-02	COL12A1
ENSG00000151748	2095.57	1.008	1.02E-06	1.52E-02	SAV1
ENSG00000137880	868.05	-1.334	1.08E-06	1.60E-02	GCHFR
ENSG00000189184	76.46	-2.031	1.12E-06	1.66E-02	PCDH18
ENSG00000180354	433.88	1.172	1.12E-06	1.67E-02	MTURN
ENSG00000137440	459.03	1.212	1.12E-06	1.67E-02	FGFBP1
ENSG00000198021	173.04	-1.717	1.14E-06	1.69E-02	SPANXA1
ENSG00000178980	1767.73	-1.030	1.16E-06	1.72E-02	SELENOW
ENSG00000130956	1126.47	1.010	1.16E-06	1.73E-02	HABP4
ENSG00000006459	694.70	1.125	1.19E-06	1.77E-02	KDM7A
ENSG00000120708	24517.44	-1.743	7.90E-19	1.17E-14	TGFBI
ENSG00000148346	38.95	2.972	1.21E-06	1.80E-02	LCN2
ENSG00000111424	593.66	-1.315	1.25E-06	1.85E-02	VDR
ENSG00000120693	82.28	2.071	1.28E-06	1.90E-02	SMAD9
ENSG00000091136	38217.09	1.012	1.30E-06	1.93E-02	LAMB1
ENSG00000255152	768.57	1.048	1.30E-06	1.93E-02	MSH5-SAPCD1
ENSG00000101082	576.50	-1.124	1.30E-06	1.94E-02	SLA2
ENSG00000101017	175.08	1.543	1.37E-06	2.04E-02	CD40
ENSG00000149633	266.28	1.319	1.38E-06	2.05E-02	KIAA1755

ENSG00000125730	785.30	-1.118	1.41E-06	2.10E-02	C3
ENSG00000168811	272.99	1.276	1.43E-06	2.13E-02	IL12A
ENSG00000157111	186.86	1.446	1.44E-06	2.14E-02	TMEM171
ENSG00000104490	24.36	-4.734	1.51E-06	2.24E-02	NCALD
ENSG00000146555	347.02	1.338	1.57E-06	2.34E-02	SDK1
ENSG00000171992	460.25	-1.633	1.62E-06	2.40E-02	SYNPO
ENSG00000154134	673.18	1.063	1.62E-06	2.41E-02	ROBO3
ENSG00000133121	1089.11	1.080	1.70E-06	2.53E-02	STARD13
ENSG00000184678	15817.52	1.057	1.72E-06	2.56E-02	HIST2H2BE
ENSG00000183117	140.06	1.560	1.72E-06	2.56E-02	CSMD1
ENSG00000139567	46.21	2.661	1.73E-06	2.57E-02	ACVRL1
ENSG00000168079	62.45	-2.186	1.73E-06	2.58E-02	SCARA5
ENSG00000141639	37.10	-3.006	1.77E-06	2.64E-02	MAPK4
ENSG00000124406	711.26	-1.180	1.79E-06	2.66E-02	ATP8A1
ENSG00000110172	4613.17	-1.047	1.82E-06	2.71E-02	CHORDC1
ENSG00000144199	289.78	1.369	1.87E-06	2.78E-02	FAHD2B
ENSG00000170175	733.90	1.095	1.89E-06	2.81E-02	CHRNB1
ENSG00000143344	1512.75	-1.037	1.93E-06	2.87E-02	RGL1
ENSG00000163235	11108.18	-1.934	3.59E-17	5.33E-13	TGFA
ENSG00000130304	947.72	1.022	2.01E-06	2.99E-02	SLC27A1
ENSG00000186575	13801.53	1.219	2.03E-06	3.02E-02	NF2
ENSG00000198963	190.84	-1.405	2.03E-06	3.02E-02	RORB
ENSG00000126091	825.92	1.024	2.04E-06	3.04E-02	ST3GAL3
ENSG00000186868	85.15	2.016	2.07E-06	3.08E-02	MAPT
ENSG00000171097	707.42	1.035	2.08E-06	3.10E-02	KYAT1
ENSG00000115738	67.86	-2.058	2.14E-06	3.19E-02	ID2
ENSG00000100767	407.81	1.144	2.20E-06	3.27E-02	PAPLN
ENSG00000145777	65.28	2.424	2.29E-06	3.41E-02	TSLP
ENSG00000139372	1264.67	-1.108	2.30E-06	3.42E-02	TDG
ENSG00000127920	1927.47	-1.135	2.32E-06	3.45E-02	GNG11
ENSG00000119866	121.95	1.740	2.32E-06	3.45E-02	BCL11A
ENSG00000171617	2424.57	-1.035	2.39E-06	3.56E-02	ENC1
ENSG00000165071	40.27	2.794	2.44E-06	3.63E-02	TMEM71
ENSG00000151623	56.63	2.287	2.53E-06	3.77E-02	NR3C2
ENSG00000186104	186.60	1.413	2.56E-06	3.80E-02	CYP2R1
ENSG00000103196	55.29	-2.690	2.56E-06	3.81E-02	CRISPLD2
ENSG00000197728	492.72	-1.100	2.61E-06	3.88E-02	RPS26
ENSG00000130283	519.41	1.285	2.69E-06	4.00E-02	GDF1
ENSG00000166292	24.74	-4.141	2.70E-06	4.02E-02	TMEM100
ENSG00000116661	225.90	-1.295	2.78E-06	4.14E-02	FBXO2
ENSG00000169946	866.03	1.006	2.79E-06	4.16E-02	ZFPM2

ENSG00000183018	374.14	1.486	2.80E-06	4.17E-02	SPNS2
ENSG00000149260	324.86	-1.263	2.84E-06	4.22E-02	CAPN5
ENSG00000136859	610.66	-1.042	2.85E-06	4.23E-02	ANGPTL2
ENSG00000163545	708.78	1.497	2.85E-06	4.24E-02	NUAK2
ENSG00000132357	321.80	-1.182	3.18E-06	4.73E-02	CARD6
ENSG00000189410	655.50	1.081	3.23E-06	4.80E-02	SH2D5

Table S2: qPCR primer sequences

Gene	Entrez-ID	forward	reverse
NGFR	NM_002507.4	ACCTCCAGAACAAGACCTCATAGC	TTGTTCTGCTTGCAGCTGTTCC
MET	NM_001127500.3	TGAAGTGGATGGCTTTGG	GCAGTATTCGGGTTGTAGG
ABCB5	NM_001163941.2	CTCACCCTAGTGACTCTATCC	CCTAAAGGCTATGACTGTTCG
CD274	NM_014143.4	CACCAATTCCAAGAGAGAGG	AGGTAGTTCTGGGATGACC
ATF3	NM_001674.4	TGTGAATGCTGAACTGAAGG	GAGGTTTCTCTCATCTTCTGG
TMEM47	NM_031442	GTACTACCTGTCGTTGTGG	CACGCAGATAGAAATCAAACC
SOX9	NM_000346	TTCATGAAGATGACCGACGAGCAGG	TCCTCGCTCTCCTTCTTCAGATCG
ACTB	NM_001101.5	GCCATGTACGTTGCTATCC	TCATGAGGTAGTCAGTCAGG
